# Establishment of IL-7 Expression Reporter Human Cell Lines, and Their Feasibility for High-Throughput Screening of IL-7-Upregulating Chemicals

**DOI:** 10.1371/journal.pone.0161899

**Published:** 2016-09-02

**Authors:** Yeon Sook Cho, Byung Soo Kim, Chan Kyu Sim, Inki Kim, Myeong Sup Lee

**Affiliations:** 1 Lab of Molecular Immunology and Medicine (MoIM), Department of Biomedical Sciences, University of Ulsan College of Medicine, Asan Medical Center, Seoul 05505, Korea; 2 Department of Convergence Medicine, Asan Institute for Life Sciences, University of Ulsan College of Medicine, Seoul 05505, Korea; Shriners Hospitals for Children, UNITED STATES

## Abstract

Interleukin-7 (IL-7) is a cytokine essential for T cell homeostasis, and is clinically important. However, the regulatory mechanism of *IL-7* gene expression is not well known, and a systematic approach to screen chemicals that regulate IL-7 expression has not yet been developed. In this study, we attempted to develop human reporter cell lines using CRISPR/Cas9-mediated genome editing technology. For this purpose, we designed donor DNA that contains an enhanced green fluorescent protein (*eGFP*) gene, drug selection cassette, and modified homologous arms which are considered to enhance the translation of the *eGFP* reporter transcript, and also a highly efficient single-guide RNA with a minimal off-target effect to target the *IL-7* start codon region. By applying this system, we established IL-7 eGFP reporter cell lines that could report *IL-7* gene transcription based on the eGFP protein signal. Furthermore, we utilized the cells to run a pilot screen campaign for IL-7-upregulating chemicals in a high-throughput format, and identified a chemical that can up-regulate *IL-7* gene transcription. Collectively, these results suggest that our IL-7 reporter system can be utilized in large-scale chemical library screening to reveal novel IL-7 regulatory pathways and to identify potential drugs for development of new treatments in immunodeficiency disease.

## Introduction

Interleukin-7 (IL-7) is a cytokine essential for T cell development and peripheral T cell homeostasis, and is mainly expressed in the epithelial and stromal cells [1–5]. *IL-7* gene expression has long been considered to be constitutive [6], but recent studies have shown that its expression can be modulated in various situations [3, 7, 8]. However, the regulatory mechanisms mediating the expression of IL-7 have not been well studied. The best known regulatory mechanism for IL-7 expression is IFN-γ-dependent, which is mediated by the interferon stimulated response element (ISRE) in the *IL-7* promoter [7, 9].

Exogenously delivered IL-7 can enhance antiviral and antitumor defense [10, 11]. Thus, IL-7 has great therapeutic potential in treating diverse immunodeficiency-related diseases [12, 13]. However, the cost of producing IL-7 protein for clinical application is much higher (>10-fold) than that of producing standard chemical drugs [14]. Therefore, identification of effective IL-7-inducing chemicals would be of great value in medicine.

To monitor IL-7 expression *in vivo*, we and others previously developed IL-7 enhanced green fluorescent protein (eGFP) or enhanced yellow fluorescent protein reporter mice [3, 4, 8, 15]. Although these reporter mice have proven to be very useful in identifying IL-7-expressing cell types [3, 5, 16], chemical screening in a high-throughput screening (HTS) format, which is very effective for identifying novel chemicals regulating specific biological pathways [17, 18] has been impractical because IL-7-expressing cells are very rare [8, 19]. Thus, establishing a more practical system such as IL-7-reporting cell lines is necessary.

Recently, CRISPR/Cas9 (Cas9)-mediated genome editing technology has proven to be very useful for introducing exogenous DNA into any locus in the genome [20–22]. Under the guidance of a single-guide RNA (sgRNA) with base-pair complementarity to a target genomic DNA (gDNA) sequence, Cas9 protein can generate site-specific DNA double-stranded breaks that promote homology-directed repair [22, 23]. Thus, any donor DNA, flanked with appropriate lengths of homology arms, can be inserted into the target locus defined by the sgRNA and Cas9 protein complex [23].

In this study, we developed a reporter system to monitor endogenous IL-7 expression in human cell lines at the transcriptional level using Cas9 gene-targeting technology. Additionally, we performed pilot screening to isolate small molecules that induced IL-7 upregulation using an HTS format and identified a polyaromatic DNA-binding small molecule that can upregulate *IL-7* gene transcription. Thus, these results suggest that our IL-7 reporter cell lines are very useful for screening IL-7-regulating chemicals in an HTS format.

## Materials and Methods

### Construction of IL-7 reporter donor DNA

**Fig 1:**
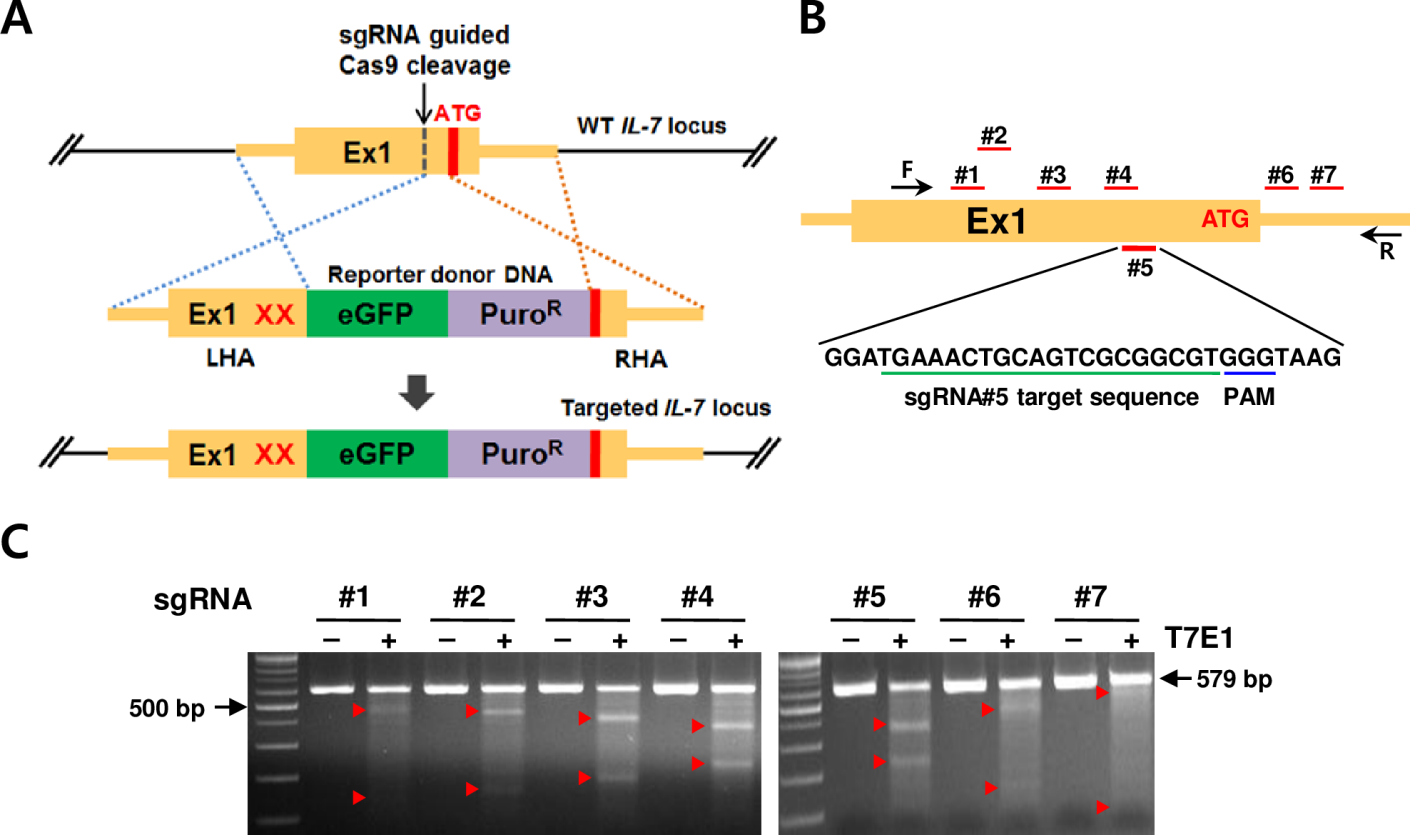
IL-7 reporter gene targeting strategy and selection of a suitable single-guide RNA (sgRNA). (A) The strategy to insert the *eGFP* reporter gene into the *IL-7* start codon region using Cas9-induced homologous recombination (HR). The insertion/target site is defined by Cas9 and sgRNA, and HR is directed by the left/right homology arms (LHA/RHA), leading to the insertion of eGFP and Puro^R^. Ex1, exon 1; ATG, authentic ATG; XX, uATG mutations (also see **S1 Fig**). (B) sgRNA targets in the *IL-7* locus are marked with red lines and the sequence for sgRNA#5 target and its associated PAM are underlined in green and blue, respectively. (C) Analysis on targeting efficiency of sgRNAs at the *IL-7* locus in 293T cells by T7E1 assay. The region covering the target sites was PCR-amplified (579 bp) with primers (F/R in black arrows in B) using gDNA of 293T cells transfected with each pX330:sgRNA, and subjected to a T7E1 assay. + means T7E1 in the reaction. Red arrowheads mark the expected fragments. Data are representative of two independent experiments.

For the construction of donor plasmid (**Fig 1A**), the right homology arm (RHA) was PCR-amplified (Phusion DNA polymerase, Thermo Scientific) and the modified left homology arm (LHA) was amplified with a two-step overlapping PCR using 293T gDNA: the first left part was amplified using primers XhoI_IL7-Left arm F/IL7 uATG mut R, and the first right part; using primers IL7 uATG mut F/HindIII_IL7-Left arm R, then, the whole arm was amplified with primers XhoI_IL7-Left arm F/HindIII_IL7-Left arm R (primer sequences in **S1 Table**). The *eGFP* expression cassette (eGFP coding sequence and SV40 polyA signal sequence) and the puromycin resistance cassette (Puro^R^) were also PCR-amplified from the pEGFP-N1 plasmid (Clontech) and the pMIR-report luciferase miRNA expression reporter (Ambion), respectively (**S1 Table**). Each PCR primer pair contained restriction enzyme site overhangs. Each fragment digested with the enzymes was sequentially inserted into pBluescript II KS (pKS, Stratagene) using XhoI-HindIII, HindIII-EcoRI, EcoRI-BamHI, and BamHI-NotI sites, producing the donor plasmid IL-7-Left arm/eGFP-SV40 polyA/Puro^R^/IL-7 right arm (pKS:IL-7 eGFP reporter donor).

### sgRNA design and pX330:sgRNA vector assembly

To design the sgRNA target sites, a 500-bp DNA sequence flanking the human *IL-7* start codon was imported into the CRISPR sgRNA design tool DNA 2.0 and Genescript [24, 25], and seven sgRNA candidates with minimal off-target effects were selected. For each target site (20-bp target sequence), a pair of oligos (**S1 Table)** annealed were inserted into the pX330 plasmid (Addgene), using the BbsI cloning site as previously described [20], to produce pX330:sgRNA#1–7.

### Human cell lines and culture

Human cell lines 293T, A549, RKO, HeLa, KATOIII, AGS, MCF-7 and PANC-1, THP-1 and Jurkat T cells were from American Type Culture Collection (ATCC, Manassas, VA) while SNU-C5 cells were from the Korean Cell Line Bank (Seoul, Korea). SNU-C5, THP-1 and Jurkat T cells were cultured in RPMI1640 containing 10% fetal bovine serum (HyClone, Logan, UT) while all other cells were cultured in Dulbecco's Modified Eagle Medium (HyClone) containing 10% fetal bovine serum at 37°C under 5% CO_2_.

### Validation of sgRNA targeting efficiency using a T7E1 assay

293T cells were transfected with each pX330:sgRNA (0.5 μg) using Lipofectamine^®^ 2000 (Invitrogen). Two days after transfection, the targeted region was PCR-amplified using gDNA from the transfected cells with the targeting test primers (**S1 Table**). The PCR products (200 ng) were denatured (95°C for 10 min) and then re-annealed. Hybridized DNA digested with T7 endonuclease I (T7E1) (1 h at 37°C) was analyzed on agarose gel.

### Generation of IL-7 eGFP reporter cell clones and genotyping

A549 and RKO cells were plated in a 6-well plate (10^6^ cells/well) and directly co-transfected with pX330:sgRNA#5 plasmid (1 μg) and the pKS:IL-7 eGFP reporter donor plasmid (1 μg) using Lipofectamine^®^ 2000 (Invitrogen) (4 μL) for 6 h. After 2.5 days, the cells from the 6-well plate were trypsinized and transferred into a 10-cm plate with 0.5 μg/mL puromycin added to the culture medium. After puromycin selection (10 days), the cells were replated in a 96-well plate (0.5 cells/well), and the selected single clones were then PCR-genotyped using the target confirmation primers, target GFP F/R and target Puro F/R (**S1 Table)**.

### Validation of the reporter clones

Selected clonal cells were treated with either IFN-γ only (50 ng/mL) for 12 h or with IFN-γ (50 ng/mL) for 12 h after 2 days of 5-azacytidine (AzaC, Sigma) treatment. *IL-7* or *eGFP* mRNA level was analyzed by quantitative reverse transcription (qRT)-PCR as described below, or the eGFP fluorescence signal was analyzed by FACS using FACSCalibur (BD Biosciences). Cell growth and sensitivity to drugs were measured as described below. To define the mutation characteristics of the on-target site guided by sgRNA#5, the targeted region was PCR-amplified using on-target F/R primers (**S1 Table**) and cloned into the T-blunt^TM^ PCR cloning vector (SolGent, Korea). Twenty clones for each cell type were sequenced and analyzed. sgRNA#5 was predicted to have only one potential off-target (TGAAACTGCAGTtGtGGgGT) with 3 bp mismatches (lower case letters) to the on-target sequence (TGAAACTGCAGTCGCGGCGT). To determine whether the potential off-target site in the cloned reporter cells was mutated, the off-target region was also PCR-amplified using off-target F/R primers (**S1 Table**) from their gDNAs, cloned, and sequenced (20 clones per reporter cell) in a manner similar to the analysis of the on-target region.

### Cell growth and drug sensitivity measurement

To measure cell growth, cells were plated onto 12-well plates (10^4^ cells/well) (D0) and the number of cells were counted every other day through day 7 (D1, D3, D5, D7). The MTT assay was used to measure the drug sensitivity of cells. For the assay, cells were plated onto a 96-well plate (10^4^ cells/well) containing phenol red-free culture media (100 μL). After 24 h, the cells were either untreated or treated with ellipticine or AzaC at the indicated doses (0.3, 0.8, 2, 5, 10 μM) for the indicated times (12, 24, 36, 48, 72 h). After removing the culture medium, the MTT assay was performed using the Vybrant MTT cell proliferation assay kit following the manufacturer’s recommendation (Invitrogen, Carlsbad, CA). Absorbance was measured at 540 nm using a microplate absorbance reader (Tecan Sunrise, Tecan Group AG, Switzerland). The drug sensitivity of cells was expressed as cell viability calculated as the ratio of the absorbance value from treated sample to that of untreated sample at each time point.

### Pilot chemical screening in a high throughput format

To determine the z′-factor (a parameter for evaluating assay feasibility), A549#6 cells were seeded onto a CELLSTAR® 96-well fluorotrac plate (Greiner Bio-One, Monroe, NC) (1.2 × 10^4^ cells/well in 100-μL culture media) under normal culture conditions. After overnight incubation, the cells were either untreated (8 wells, negative control) or treated (8 wells, positive control) with media containing 5 μM AzaC for 2 days and then with IFN-γ (50 ng/mL) for 16 h. For pilot-screening, A549#6 cells were plated onto 96-well plates and treated with AzaC for two days in a similar manner as described above. The cells were then further treated for 16 h with media containing the library compound (10 μM of final compound in 0.1% final DMSO) delivered using an automatic liquid handler (Janus, Perkin Elmer, Waltham, MA). The treated cells were then stained with Hoechst 33342 dye in HBSS buffer (20 min at 37°C, 5% CO_2_). The eGFP signal in live cells was imaged (20× and Alexa488 channel) using an Operetta High Contents Screen system (PerkinElmer), and the mean eGFP signal intensity per well was quantified using Harmony software (Perkin Elmer). The z′-factor was calculated from the signals of positive and negative controls as described previously [26]. Using this method, 400 compounds in the LOPAC1280^TM^ library were pilot-screened in five 96-well plates containing negative controls (8 wells of untreated A549#6 cells per plate) and positive controls (8 wells of IFN-γ + AzaC treated A549#6 cells per plate). The z-factor of each plate was calculated from the signals of the controls and samples as previously reported [26]. A ‘hit’ compound was defined as a compound showing a more than 1.5-fold increase in GFP signal compared to the mean GFP value from all wells.

### Gene expression analysis by qRT-PCR

Total RNAs purified from cells using QIAzol RNA isolation reagents (Qiagen) were reverse-transcribed into cDNAs using SuperScript II reverse transcriptase (Invitrogen). The expression levels of individual genes were measured by quantitative PCR using the CFX Connect Real-Time PCR detection system (Br185-5200, Bio-Rad) as previously described [27], using gene-specific forward (F) and reverse (R) primers (**S2 Table**). The mRNA expression level of each gene was normalized to that of *GAPDH*.

### IL-7 protein analysis by enzyme-linked immunosorbent assay (ELISA)

To measure IL-7 protein levels, cells (10^4^/well) were plated onto 96-well plates and either untreated or treated with ellipticine (5 μM) for 24 or 36 h. The culture supernatant was collected and IL-7 protein level was measured using the Human IL-7 Quantative HS ELISA Kit following the manufacturer’s instructions (R&D Systems, Minneapolis, MN).

### Statistical analysis

All data are presented as mean ± standard deviation. All the statistical analyses were performed using a two-tailed unpaired Student’s *t* test. *P* < 0.05 was considered statistically significant.

## Results

### Design of IL-7 eGFP reporter donor DNA

We first designed the donor DNA template for enhancing the translation of *eGFP* transcripts in the *IL-7* locus. The overall donor DNA structure included the left homologous arm (LHA; 1083 bp), *eGFP* coding sequence with a poly A signal sequence (eGFP), puromycin selection cassette (Puro^R^), and right homologous arm (RHA; 1072 bp) (**Fig 1A**). The best insertion position of the eGFP reporter would be at the start codon of the *IL-7* gene. However, the *IL-7* 5′ untranslated region (UTR) contains several upstream ATGs (uATGs), which are known to inhibit the translation of the authentic ATG start codon in *IL-7* and many other mRNAs [28–31]. Thus, eGFP protein production from *eGFP* mRNA containing uATGs would be very weak, even though the *eGFP* mRNA exists. To avoid this potential problem, we intentionally designed the LHA to remove several uATGs by not including part of the 5′ UTR and mutating the uATGs, without affecting *IL-7* promoter sequences such as the ISRE (**S1 Fig**) which can respond to IFN-γ, and designed the RHA to start from the authentic ATG codon [9, 32]. This strategy is similar to what we previously used successfully to produce IL-7 eGFP reporter mice [8].

### Selection of a suitable sgRNA for inserting the IL-7 reporter DNA into the IL-7 start codon region

To introduce the constructed eGFP reporter donor into a defined site of the genome using Cas9 protein, we first chose seven sgRNAs that target sequences near the start codon of the *IL-7* gene with a minimal off-target effect (**Fig 1B** and **S1 Table**), and inserted each annealed sgRNA oligo into the pX330 plasmid, which expresses Cas9 protein and sgRNA. To test the targeting efficiency of the sgRNAs, each Cas9:sgRNA plasmid was transfected into 293T cells, and 293T gDNA was analyzed by a T7 endonuclease I (T7E1) assay. The assay showed that sgRNAs #3, #4, and #5 could highly efficiently induce mutation in the target loci (**Fig 1C**). Considering the potential off-target number, we chose sgRNA#5 (containing only one potential off-target) which targets a sequence in the 5′ UTR as the optimal sgRNA.

### Establishment of human IL-7 reporter cell lines

**Fig 2:**
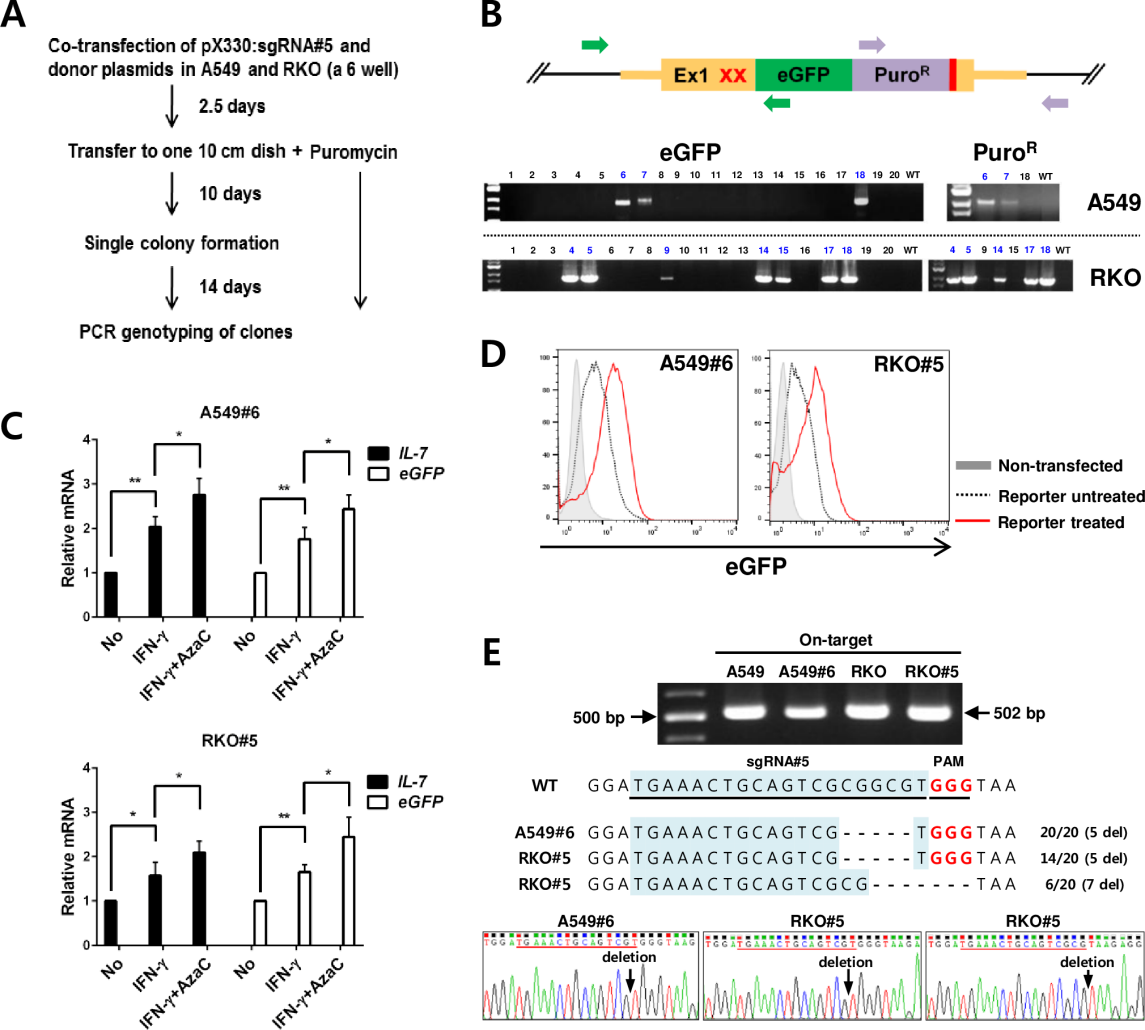
Establishment of IL-7 eGFP reporter cell lines. (A) Scheme for generating IL-7 eGFP reporter clones from A549 and RKO cells. (B) A representative result of PCR-genotyping (among more than 100 clones from each cell) to identify the correct insertion of *eGFP* (left) and Puro^R^ (right) in the *IL-7* locus of single clones of targeted A549 and RKO cells. Primers (green and purple arrows) to amplify the eGFP insertion (967 bp) and Puro^R^ insertion (1423 bp) were indicated on the diagram (top), and PCR products were shown in the gels (bottom). (C) Selected clones (A549#6 and RKO#5) were untreated (No), treated with IFN-γ alone (12 h), or treated with IFN-γ (12 h) after 2 days of AzaC treatment (5 μM for A549#6 and 2 μM for RKO#5) (n = 4 per group), and *IL-7* and *eGFP* mRNA expression was analyzed by qRT-PCR. Data are representative of two independent experiments. (D) Non-transfected cells (A549 and RKO) and reporter clones (A549#6 and RKO#5) were either untreated (reporter, untreated) or treated with IFN-γ (16 h)/AzaC (2 days) as described in C, and their eGFP signals were analyzed by FACS. Data are representative of two independent experiments. (E) On-target site analysis. On-target regions from the reporter clones were PCR-amplified, cloned and sequenced (20 clones). Agarose gel image of PCR products (top). Sequences from original cells (WT) and reporter clones (A549#6 and RKO#5) (middle). All 20 sequences from A549#6 contained a 5-base deletion (marked as -) in the target site while 14 and 6 sequences from RKO#5 contained 5-base and 7-base deletions, respectively. sgRNA and PAM sites are underlined. Representative chromatograms showing the deletions in the read sequences (bottom). *P < 0.05, **P < 0.01.

Since IL-7 has been reported to be expressed in the epithelial cells of the lung and intestine [3, 4, 8], we chose two human cell lines, A549 (lung epithelial cells) and RKO (colon epithelial cells), as recipient cells. We next introduced the selected pX330:sgRNA#5 with the IL-7 eGFP reporter donor template into these cells by transfection, and selected puromycin-resistant single clones (**Fig 2A**).

To confirm the correct gene targeting, the expanded single clones were PCR-genotyped sequentially to detect the insertion of *eGFP* and then that of Puro^R^ in the correct position (**Fig 2B**). The correct *eGFP* targeting efficiency was 15–42%, while the correct Puro^R^ targeting efficiency among eGFP-targeted cells was greater than 60% (**Fig 2B**). Accordingly, we could obtain more than four correctly targeted A549 and RKO cells, respectively, from one transfection.

To select the best clones that report IL-7 expression, we decided to treat the genotype-confirmed clonal cells with IFN-γ (a known IL-7 inducer) alone or with IFN-γ and AzaC (a DNA methylation inhibitor) [33, 34]. AzaC was included because the *IL-7* promoter region contains a CpG island, and methylated CpG could suppress *IL-7* gene transcription (**S1 Fig**) [35, 36]. The dose of AzaC that did not cause significant cytotoxicity after several days of treatment was ≤ 5 μM for A549 and ≤ 2 μM for RKO (**S2A** and **S2B Fig**). AzaC alone induced low IL-7 expression in A549 cells but not in RKO cells after treatment (**S2C** and **S2D Fig**). When reporter cells were tested for IL-7 induction, *IL-7* mRNA was induced by IFN-γ treatment as expected, and was further induced by the addition of AzaC (**Fig 2C**). The clones A549#6 and RKO#5 showed good correlative expression of *IL-7* and *eGFP* mRNAs (**Fig 2C**). These selected reporter cells and original cells showed comparable growth rates, cell morphology, and sensitivity to AzaC, as well as a comparable IL-7 response to IFN-γ (**S2–S4 Figs**). Additionally, these reporter cells responded well to IFN-γ and AzaC co-treatment at the eGFP protein level, as determined by FACS analysis (**Fig 2D**). The selected reporter cells appeared to contain both the eGFP-inserted allele in the IL-7 loci (**Fig 2B**) and correct sgRNA-targeted mutated allele containing a short deletion in the IL-7 5′ UTR (**Fig 2E**), which did not appear to affect IL-7 protein production (see below). However, these reporter cell lines seemed not mutated at the potential off-target locus possibly inducible by Cas9 protein (**S4B Fig**). Together, these results indicate that our current approach led to the successful generation of IL-7 eGFP reporter cell lines.

### Pilot screening for chemicals inducing IL-7 gene upregulation in an HTS format

**Fig 3:**
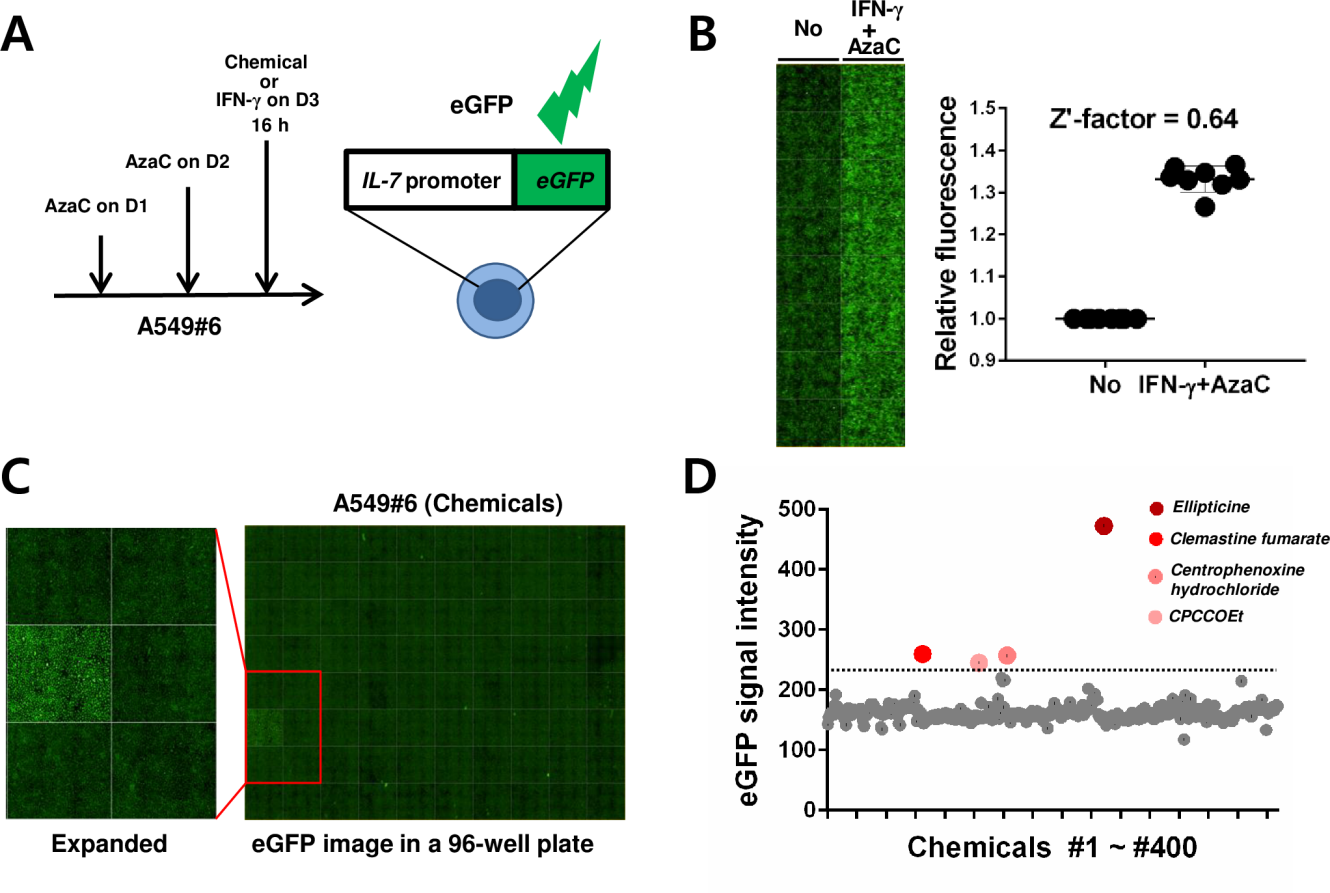
Pilot screening for chemicals inducing *IL-7* gene upregulation in an HTS format. (A) Scheme for chemical screening. A549#6 cells in a 96-well plate were treated with AzaC for 2 days and then with IFN-γ or chemicals for 16 h. (B) HTS assay feasibility test. A549#6 cells plated on 96-wells were either untreated (8 wells, negative control) or treated (8 wells, positive control) with 5 μM AzaC for 2 days and then with IFN-γ (50 ng/mL) for 16 h (IFN-γ + AzaC). The eGFP signals were imaged (left panel) and quantified; the quantified signals from treated samples normalized to those of untreated samples were plotted and the calculated z′-factor is shown (right panel). Data are representative of two independent experiments. (C) A representative eGFP image from A549#6 cells treated with 80 chemicals in a 96-well plate: the hit is ellipticine. (D) Quantitated eGFP signal intensity for wells treated with 400 chemicals in 96-well plates. The horizontal line marks a 1.5-fold increase above the mean signal intensity.

Since the IL-7 reporter clone A549#6 reflected *IL-7* transcription well and attached better than RKO cells, we used this clone for testing the feasibility of screening IL-7-upregulating chemicals in an HTS format. To check if the system works in a 96-well format, the reporter cells were either untreated or first treated with AzaC and then with IFN-γ in a 96-well plate, and their eGFP signals were imaged (**Fig 3A**). As expected, the treatment led to a considerable enhancement in the eGFP signal, showing a z′-factor (assay feasibility index) of 0.64 (**Fig 3B**), indicating that our assay is effective [26]. Using this scheme, we pilot-screened 400 chemicals in a 96-well format, and found that four of the chemicals induced eGFP signals more than 1.5-fold above average (Fig **3C** and **3D**). The quality of screening was good because it showed reasonable z-factors (range: 0.4–0.6) [26]. Among the screened chemicals, ellipticine (EL), a multi-target prodrug [37], showed the greatest eGFP signal (~3.0-fold) (**Fig 3C** and **3D**). Because other chemicals showed very marginal induction, we focused only on EL for reconfirmation below.

### Confirmation of a screened chemical that upregulated IL-7 expression

**Fig 4:**
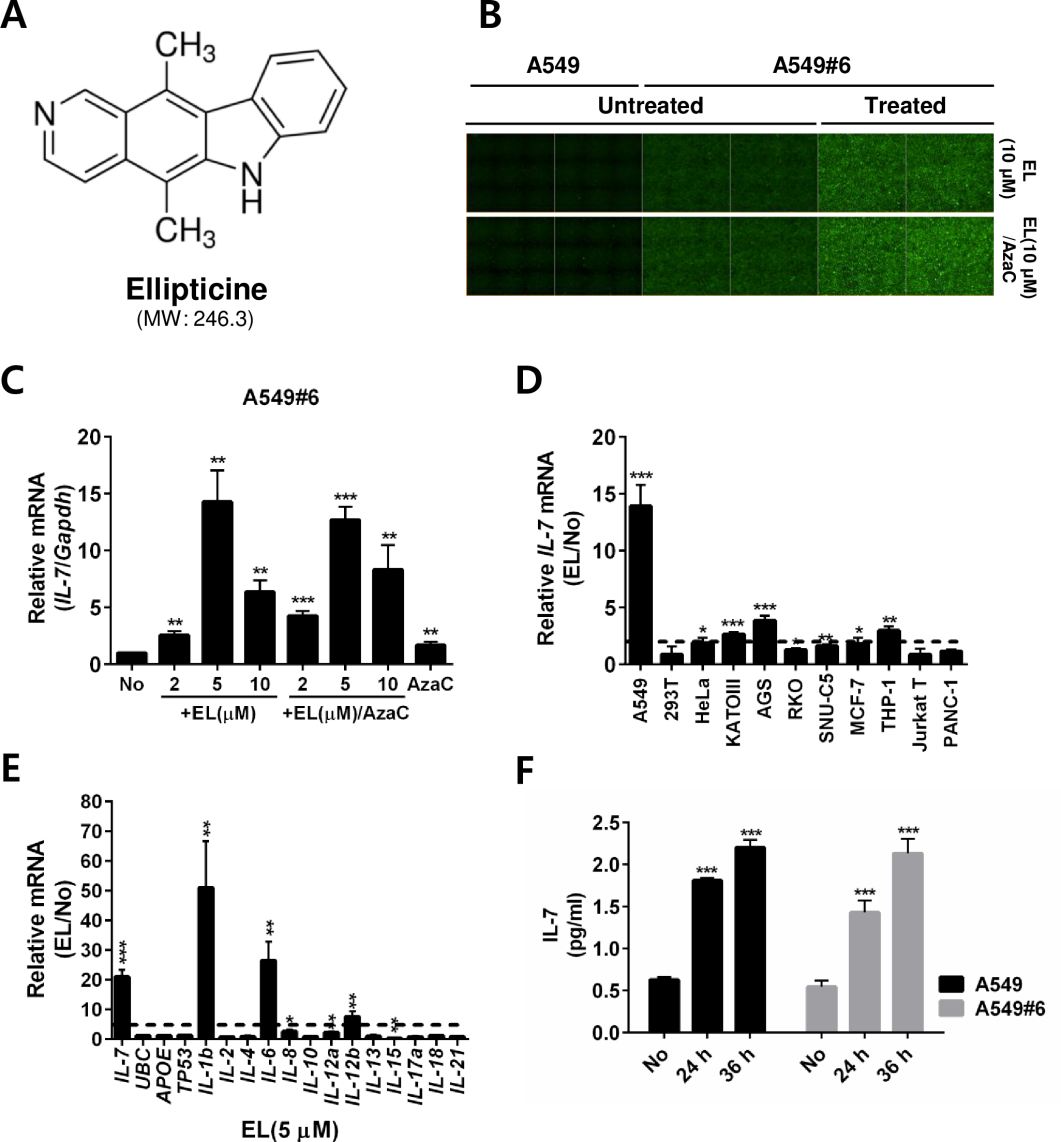
Confirmation of a screened chemical upregulating IL-7 expression. (A) Chemical structure of ellipticine. (B, C) A549 or A549#6 cells were untreated (No), and A549#6 cells were treated with ellipticine (EL, 10 μM for B) alone or EL for 16 h after treatment with AzaC (5 μM) for 2 days (B), or with EL alone (12 h) (C), with EL for 12 h after treatment with AzaC for 2 days (C) or with AzaC alone for 3 days (C) (n = 4 per group). The eGFP image (B) was captured and *IL-7* mRNA expression (C) was quantified by qRT-PCR; signals from treated samples were compared to those of untreated samples (No) for statistical analysis and calculating fold-induction. (D) Various human cell lines were untreated (No) or treated with EL (5 μM) (n = 4 per group) for 12 h and the *IL-7* mRNA expression levels were measured by qRT-PCR. The IL-7 signal from the EL-treated sample (EL) was normalized to that from the untreated sample (No) and the results are shown as relative *IL-7* mRNA. Dashed line indicates 2-fold induction. (E) A549 cells (n = 4 per group) treated with EL as described in D were analyzed for mRNA expression levels for various interleukins and several genes commonly evaluated in the literature (*UBC*, *APOE* and *TP53*). The signal from the EL-treated sample (EL) was normalized to that of the untreated sample (No) and the results are shown as relative mRNA. Dashed line indicates 5-fold induction. (F) IL-7 protein analysis by ELISA. A549 and A549#6 cells were either untreated (No) or treated (n = 4 per group) with EL (5 μM) for 24 or 36 h and the culture supernatant was analyzed by ELISA. Data are representative of at least two independent experiments. *P < 0.05, **P < 0.01, ***P < 0.001.

To test whether the top selected chemical, EL (**Fig 4A**), could actually induce *IL-7* gene transcription, we treated the clone A549#6 with EL with or without AzaC. As expected, EL induced the eGFP signals, even without AzaC treatment (**Fig 4B**), and also induced *IL-7* mRNA expression (**Fig 4C**), indicating that EL indeed induces *IL-7* gene upregulation. Since *IL-7* induction is strongest at 5 μM and the cytotoxicity at this dose is very mild (**S5 Fig**), this concentration was used in the following experiment. We measured the EL-dependent *IL-7* induction response in various cell lines and found that the degrees of response were quite different in different cell types. The strongest response was observed in A549 cells, while no response was observed in 293T and Jurkat T cells (**Fig 4D**). Similarly, only a few genes, such as *IL-1b*, *IL-6*, and *IL-12b*, among popular interleukin genes tested were strongly induced in A549 cells upon EL treatment, while other interleukins were not induced or were only mildly induced (**Fig 4E**). Additionally, several genes for common proteins (UBC, APOE, and TP53) in the literature were not induced (**Fig 4E**). These results indicate that EL induces a specific subset of interleukins, including IL-7, in A549 cells. Consistent with the mRNA expression results, the IL-7 protein level measured by ELISA was upregulated in A549 cells upon EL treatment (**Fig 4F**). EL-treated A549#6 reporter cells also showed a similar level of IL-7 protein (**Fig 4F**), indicating that the targeted deletion in the *IL-7* 5′ UTR in the reporter cells (**Fig 2E**) did not affect the *IL-7* mRNA expression and translation of the IL-7 coding sequence. Collectively, these results indicate that A549#6, and thus our reporter strategy/system, is very useful for screening IL-7-upregulating chemicals in HTS formats.

## Discussion

In this study, we describe, for the first time, the development of an IL-7 eGFP reporter cell line using CRISPR/Cas9 genome-editing technology, which reports the *IL-7* transcription level as an eGFP protein signal. Additionally, we show that the reporter system can be used to screen chemicals inducing *IL-7* gene upregulation in an HTS format. Although our HTS design for identifying small molecules that upregulate endogenous target gene transcription is not commonly used [38, 39], our screening scheme and pilot screening performance was found to be good and robust; we obtained a good z′-factor (HTS assay feasibility index) and z-factors (HTS quality index) [26] and identified the EL, an IL-7 upregulator, through small-scale screening. In addition, we found that EL upregulated *IL-7* gene transcription strongly in A549 lung cells and weakly in several other cancer cell lines. However, EL also induced the expression of several other proinflammatory cytokine genes, indicating that EL is not an IL-7-specific inducer. Since EL is a prodrug and its effect on cell physiology depends on the expression/function of multiple proteins such as cytochrome P450, peroxidases, and vacuolar (V)-ATPases, the mechanism by which the prodrug upregulates the expression of IL-7 and other interleukins remains unclear [37, 40, 41].

Although we demonstrated the feasibility of using the reporter cells for screening chemicals to upregulate *IL-7* transcription, the reporter cells could also be used to screen chemicals that downregulate *IL-7* transcription in an HTS format. Such chemicals could be identified by determining the wells showing a weaker eGFP signal than non-treated wells and selecting those in which chemical-treated cells are healthy (easily determined by cell imaging). Additionally, the reporter cells could be effectively used to identify *IL-7*-upregulating genes at the genome-wide level. For example, a random expression cDNA library can be produced from the high IL-7-expressing cells, and then introduced into the IL-7 reporter cells by infection. Then, the cells showing higher eGFP expression than uninfected cells can be sorted by FACS, and their cDNAs can be cloned using the known sequence in the delivered vector. Since only positive clones are sorted based on the eGFP signal, the process is not labor-intensive but nonetheless effective [42].

Many mRNAs in the human genome contain uATGs in their 5′ UTRs [29–31], which suppress the translation of the downstream authentic ATG. In such cases, HTS screening for transcriptional upregulators or expression-inducing chemicals using a transcriptional reporter is not an easy task. Thus, as demonstrated in this study, the proposed strategy of inserting an *eGFP* sequence in the authentic start codon of the target gene after mutating such uATGs in the 5′ UTRs within the LHA can be applied to the system. Because these reporter transcripts are efficiently translated into the fluorescent eGFP protein, the activity can be read automatically in an HTS format.

The promoter regions of many genes contain CpG islands, which are methylated in cell lines, and their compact chromatin structure inhibits their efficient transcription [35, 36]. This feature of many cancer cells might pose a challenge in screening for positive regulating factors and chemicals; that is, even if a reporter gene is successfully inserted into the endogenous locus, the gene may be transcriptionally silenced. In the current study, we used AzaC treatment before performing the chemical treatment, which increased the IL-7 induction efficiency. Thus, we consider that treating certain cells with demethylating agents such as AzaC would facilitate screening for transcription-regulating chemicals of a target gene containing methylated CpG DNA.

In future, it would be valuable to screen large chemical libraries to identify chemicals that specifically upregulate *IL-7* gene transcription using the established IL-7 eGFP reporter cells. Additionally, it would be interesting to explore the biological mechanism of IL-7 upregulation by the identified chemicals and their biological functions *in vivo*.

## Supporting Information

S1 Fig Left homologous arm sequence and feature. Interferon-stimulated response element (ISRE) is indicated in yellow background. CpG island (annotated in the UCSC genome browser) is present in the promoter region and the 1^st^ intron covering 722 bp (the start site is marked by a black triangle). Upstream ATGs (uATGs) are shown in red letters and the authentic ATG start site is shown in red letters over a green background. Mutated uATGs (T to G) are marked by Xs and the deleted part of the 5′ UTR is shown by gray shading. The right homologous arm starts from the authentic ATG codon extending to the 1^st^ intron.
(TIF)

S2 Fig Cytotoxicity and *IL-7* inducibility of AzaC in lung and colon cancer cells. (A,B,E,F) Cytotoxicity test. A549 (A) and RKO (B) cells as well as A549#6 (E) and RKO#5 cells (F) were either untreated (No) or treated (n = 3 per group) with the indicated concentrations of AzaC for up to 72 h. Cell viability of the treated cells was measured using the MTT assay and the results are shown as the signal from the AzaC-treated sample normalized to that of the untreated sample at each time point. (C,D) *IL-7* inducibility by AzaC. A549 (C) and RKO (D) cells were either untreated (No) or treated (n = 4 per group) with the indicated concentrations of AzaC for up to 3 days (D1, D2, D3). The *IL-7* mRNA level from the AzaC-treated sample (AzaC) was normalized to that from the untreated sample (No) and shown as relative *IL-7* mRNA. Data are representative of two independent experiments. *P < 0.05, **P < 0.01.
(TIF)

S3 Fig Growth curve and cell morphology are not significantly different between original cells and IL-7 eGFP reporter cells. (A, B) A549 and A549#6 cells as well as RKO and RKO#5 cells (10^4^ cells per 12 well, n = 4 per group) were plated (D0) and the number of cells was counted every other day through day 7 (D1, D3, D5, D7). The A549 and A549#6 cell growth curve (A) and RKO and RKO#5 cell growth curve (B) are shown. (C, D) The cells were plated onto six wells (10^5^/well) and photographed at 36 h to observe cell morphology. A549 and A549#6 cell images (C) and RKO and RKO#5 cell images (D) are shown. Scale bar is 50 μm.
(TIF)

S4 Fig *IL-7* expression comparison between original cells and reporter cells as well as potential off-target site analysis in reporter cells. (A) *IL-7* expression comparison. A549 and A549#6 cells as well as RKO and RKO#5 cells were either untreated (No) or treated (n = 4 per group) with IFN-γ (50 ng/mL) alone for 12 h or IFN-γ (50 ng/mL) for 12 h after treatment with AzaC for 2 days (5 μM for A549s, 2 μM for RKOs). *IL-7* expression levels were measured by qRT-PCR. The signals from reporter cells (A549#6 and RKO#5) were normalized to those from original cells (A549 and RKO), respectively. (B) Potential off-target site analysis on reporter cells. The genomic DNAs from A549#6 and RKO#5 cells were PCR-amplified using on-target confirmation primers (on-target F/R primers). PCR products were then cloned and 20 independent clones were sequenced and analyzed for potential mutations. Agarose gel image of the PCR products (top). Sequence of on-target and potential off-target site (middle). None of the 20 sequences from A549#6 and RKO#5 contained mutation in the potential off-target site. sgRNA and PAM sites are underlined. Representative chromatograms of the read sequences from clones derived from both cell types (bottom).
(TIF)

S5 Fig Cytotoxicity test for ellipticine on A549 and A549#6 cells. A549 (A) and A549#6 (B) cells (10^4^/well) plated on 96-wells were either untreated (No) or treated (n = 3 per group) with the indicated concentration of ellipticine for up to 36 h. Cell viability of the treated cells was measured using the MTT assay and the results are shown as the signal from the ellipticine-treated sample normalized to that from the untreated sample at each time point. Data are representative of two independent experiments. *P < 0.05, **P < 0.01, ***P < 0.001.
(TIF)

S1 Table DNA primers used for reporter cell establishment. (XLSX)

S2 Table DNA primers used for qRT-PCR analysis. (XLSX)

## Abbreviations

eGFP: enhanced green fluorescent protein
FACS: fluorescence activated cell sorting
gDNA: genomic DNA
HTS: high-throughput screening
IL-7: interleukin-7
PCR: polymerase chain reaction
sgRNA: single-guide RNA
