# Supplementary figures and images for: Establishment of IL-7 Expression Reporter Human Cell Lines, and Their Feasibility for High-Throughput Screening of IL-7-Upregulating Chemicals
Source: PLoS One. 2016 Sep 2;11(9):e0161899. doi: 10.1371/journal.pone.0161899 (PMC5010243; doi:10.1371/journal.pone.0161899)

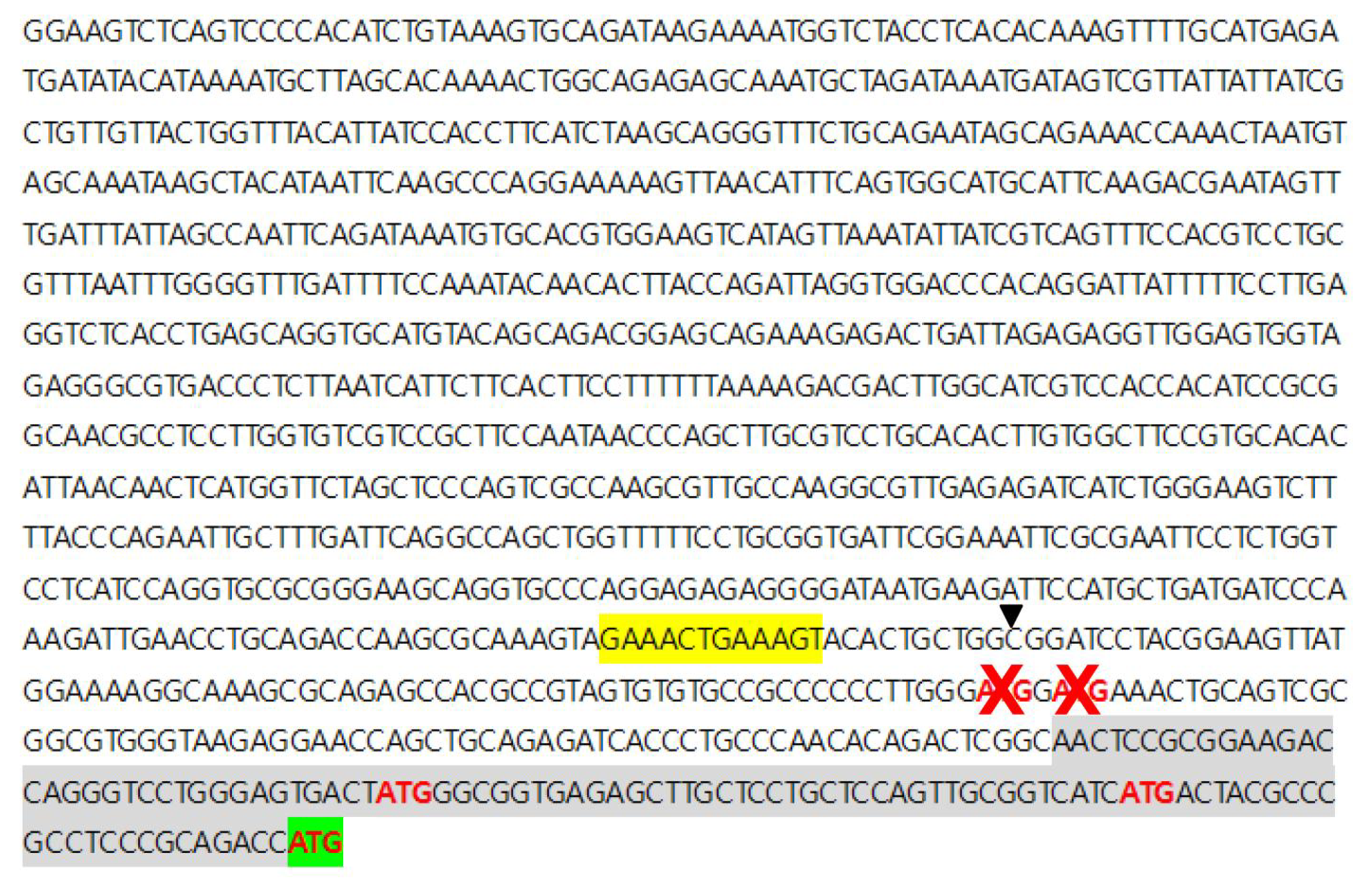

Supplement: S1 Fig — Interferon-stimulated response element (ISRE) is indicated in yellow background. CpG island (annotated in the UCSC genome browser) is present in the promoter region and the 1st intron covering 722 bp (the start site is marked by a black triangle). Upstream ATGs (uATGs) are shown in red letters and the authentic ATG start site is shown in red letters over a green background. Mutated uATGs (T to G) are marked by Xs and the deleted part of the 5′ UTR is shown by gray shading. The right homologous arm starts from the authentic ATG codon extending to the 1st intron. (TIF) [file pone.0161899.s001.tif]

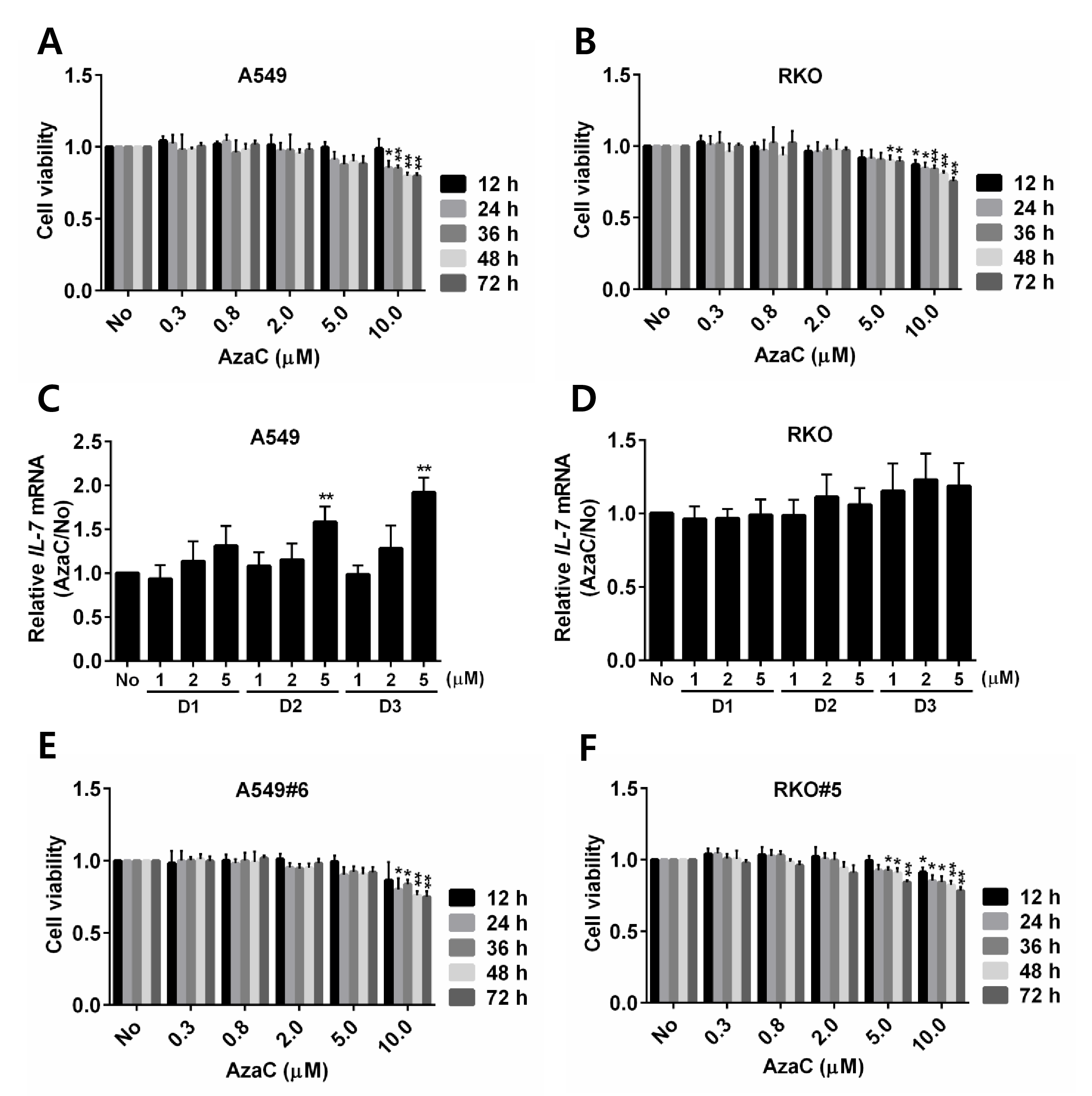

Supplement: S2 Fig — (A,B,E,F) Cytotoxicity test. A549 (A) and RKO (B) cells as well as A549#6 (E) and RKO#5 cells (F) were either untreated (No) or treated (n = 3 per group) with the indicated concentrations of AzaC for up to 72 h. Cell viability of the treated cells was measured using the MTT assay and the results are shown as the signal from the AzaC-treated sample normalized to that of the untreated sample at each time point. (C,D) IL-7 inducibility by AzaC. A549 (C) and RKO (D) cells were either untreated (No) or treated (n = 4 per group) with the indicated concentrations of AzaC for up to 3 days (D1, D2, D3). The IL-7 mRNA level from the AzaC-treated sample (AzaC) was normalized to that from the untreated sample (No) and shown as relative IL-7 mRNA. Data are representative of two independent experiments. *P < 0.05, **P < 0.01. (TIF) [file pone.0161899.s002.tif]

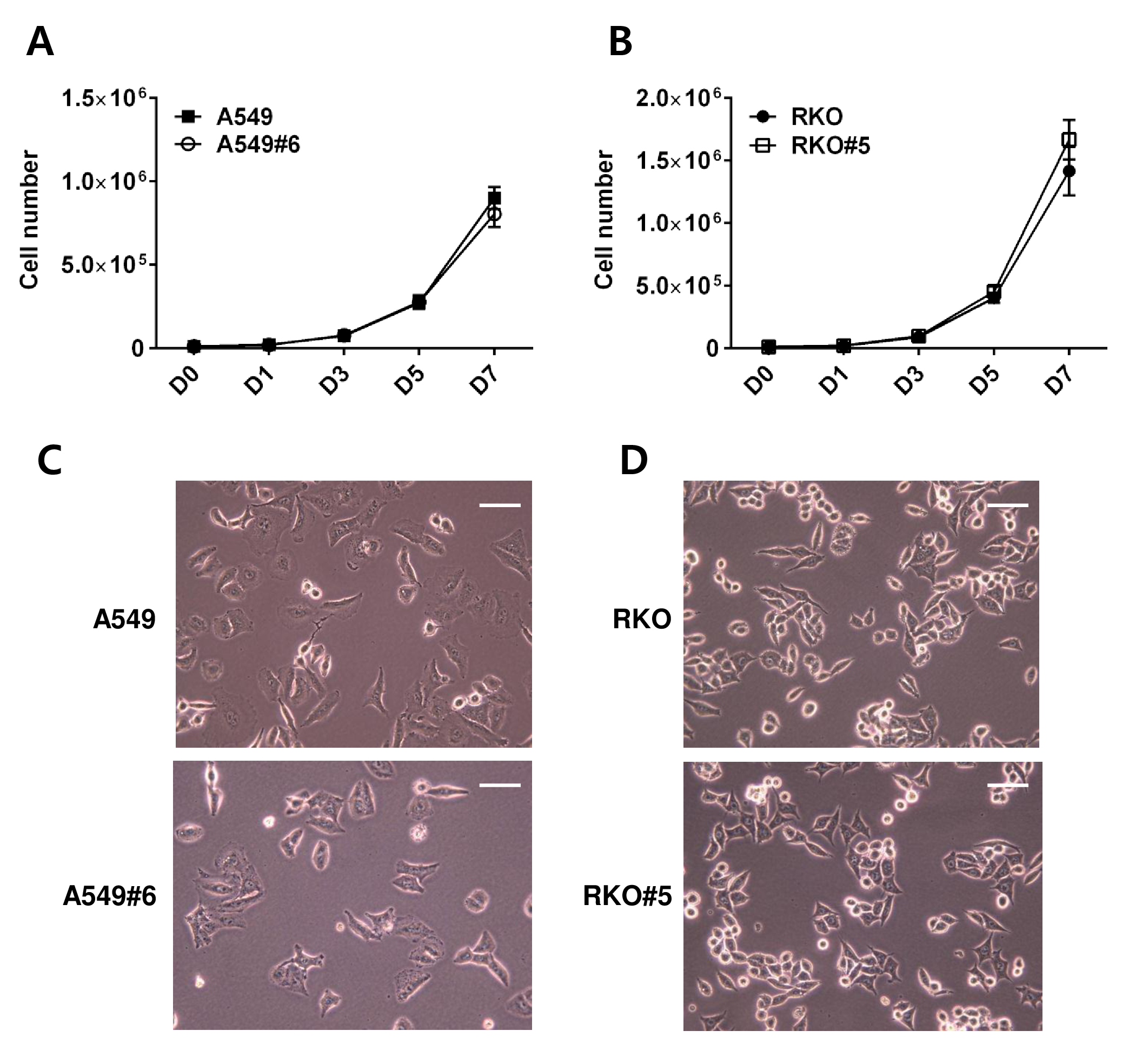

Supplement: S3 Fig — (A, B) A549 and A549#6 cells as well as RKO and RKO#5 cells (104 cells per 12 well, n = 4 per group) were plated (D0) and the number of cells was counted every other day through day 7 (D1, D3, D5, D7). The A549 and A549#6 cell growth curve (A) and RKO and RKO#5 cell growth curve (B) are shown. (C, D) The cells were plated onto six wells (105/well) and photographed at 36 h to observe cell morphology. A549 and A549#6 cell images (C) and RKO and RKO#5 cell images (D) are shown. Scale bar is 50 μm. (TIF) [file pone.0161899.s003.tif]

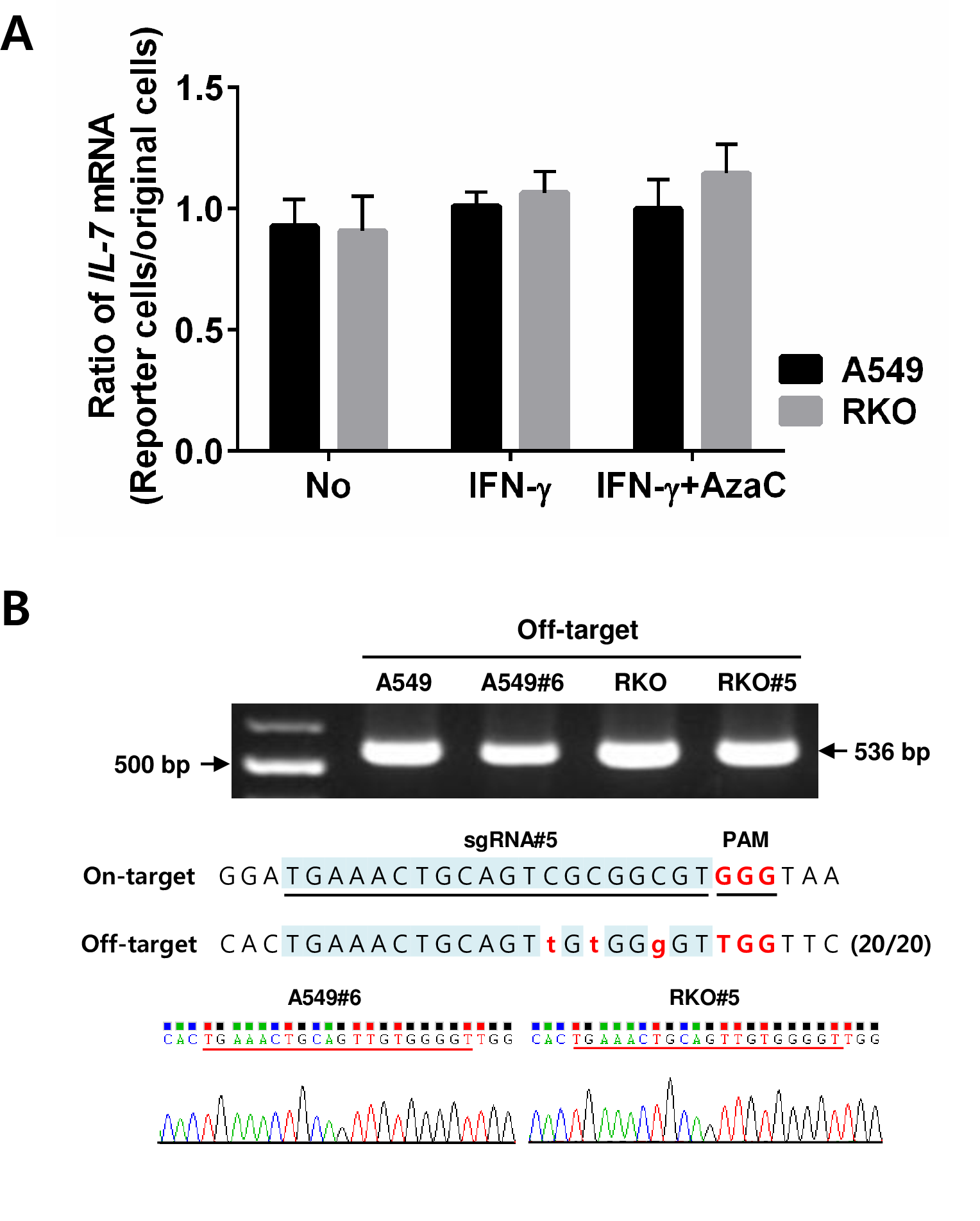

Supplement: S4 Fig — (A) IL-7 expression comparison. A549 and A549#6 cells as well as RKO and RKO#5 cells were either untreated (No) or treated (n = 4 per group) with IFN-γ (50 ng/mL) alone for 12 h or IFN-γ (50 ng/mL) for 12 h after treatment with AzaC for 2 days (5 μM for A549s, 2 μM for RKOs). IL-7 expression levels were measured by qRT-PCR. The signals from reporter cells (A549#6 and RKO#5) were normalized to those from original cells (A549 and RKO), respectively. (B) Potential off-target site analysis on reporter cells. The genomic DNAs from A549#6 and RKO#5 cells were PCR-amplified using on-target confirmation primers (on-target F/R primers). PCR products were then cloned and 20 independent clones were sequenced and analyzed for potential mutations. Agarose gel image of the PCR products (top). Sequence of on-target and potential off-target site (middle). None of the 20 sequences from A549#6 and RKO#5 contained mutation in the potential off-target site. sgRNA and PAM sites are underlined. Representative chromatograms of the read sequences from clones derived from both cell types (bottom). (TIF) [file pone.0161899.s004.tif]

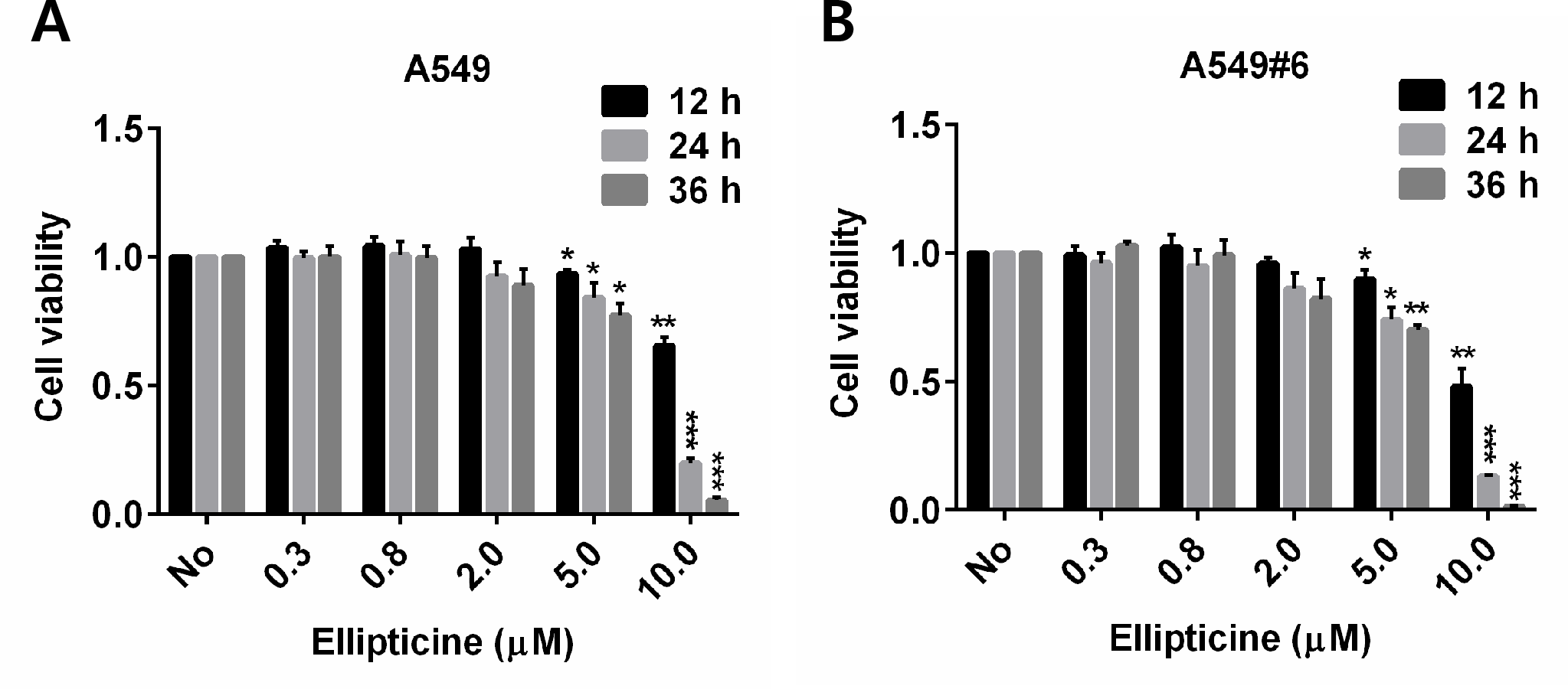

Supplement: S5 Fig — A549 (A) and A549#6 (B) cells (104/well) plated on 96-wells were either untreated (No) or treated (n = 3 per group) with the indicated concentration of ellipticine for up to 36 h. Cell viability of the treated cells was measured using the MTT assay and the results are shown as the signal from the ellipticine-treated sample normalized to that from the untreated sample at each time point. Data are representative of two independent experiments. *P < 0.05, **P < 0.01, ***P < 0.001. (TIF) [file pone.0161899.s005.tif]
